# Phagenendolysine – eine neue Wirkstoffklasse mit vielfältigen Einsatzmöglichkeiten

**DOI:** 10.1007/s00103-025-04059-9

**Published:** 2025-05-06

**Authors:** Evgeny A. Idelevich, Karsten Becker

**Affiliations:** 1 Friedrich Loeffler-Institut für Medizinische Mikrobiologie, Universitätsmedizin Greifswald, Ferdinand-Sauerbruch-Str., 17475 Greifswald, Deutschland; 2 Institut für Medizinische Mikrobiologie, Universitätsklinikum Münster, Münster, Deutschland

**Keywords:** Bakteriophage, Endolysin, Infektion, Therapie, Prävention, Bacteriophage, Endolysin, Infection, Therapy, Prevention

## Abstract

Endolysine stellen als „Enzybiotika“ eine neue Klasse antibakterieller Wirkstoffe dar, die natürlicherweise am Ende des lytischen Zyklus in den Bakteriophagen-infizierten Bakterienzellen produziert werden, um den gebildeten Phagenpartikeln vom Zellinneren heraus die Freisetzung aus der Wirtszelle zu ermöglichen. Ihre enzymatische Wirkung auf das Zellwandpeptidoglykan, die zur Lyse der befallenen Bakterien führt, können sie als applizierte Substanz auch von außen entfalten. Während die Endolysinaktivität bei grampositiven Bakterien direkt wirksam werden kann, muss bei gramnegativen Bakterien das Endolysin so modifiziert werden, dass es die äußere Zellmembran überwinden kann. Deshalb sowie zur Optimierung ihrer Spezifität und Stabilität werden Endolysine zunehmend gentechnisch modifiziert und rekombinant produziert, was aufgrund ihres modularen Aufbaus aus lytischen und Bindedomänen relativ einfach realisierbar ist. Bereits jetzt haben Endolysine zunehmend einen tatsächlichen oder umfangreich postulierten Einsatz zu präventiven, therapeutischen und diagnostischen Zwecken in der Human- und Veterinärmedizin sowie in der Lebensmittelsicherheit, Biotechnologie und im One-Health-Sektor gefunden, den es jedoch durch valide Studien noch besser zu belegen gilt. Obwohl die regulativen Aspekte im Gegensatz zur Phagentherapie entsprechend den auch für andere Arzneimittel und Medizinprodukte vorgeschriebenen Zulassungsverfahren folgen können, sind im humanmedizinischen Bereich erst weniger als ein Dutzend randomisierte kontrollierte Studien der Phasen 1 bis 3 initiiert oder abgeschlossen worden. Marktverfügbar sind bisher nur sehr wenige, als Medizinprodukt zugelassene Endolysinformulierungen und für einige Endolysine wird die Zulassung als Arzneimittel angestrebt.

## Abstract (en)

As “enzybiotics,” endolysins represent a new class of antibacterial agents that are naturally produced at the end of the lytic cycle in bacteriophage-infected bacterial cells to enable the phage particles formed to be released from the inside of the host cell. Their enzymatic effect on the cell wall peptidoglycan, which leads to lysis of the infected bacteria, can also be exerted externally as an applied agent. While the endolysin activity can be directly effective in Gram-positive bacteria, the endolysin must be modified for activity against Gram-negative bacteria so that it can overcome the outer cell membrane. For this reason, and to optimize endolysin specificity and stability, endolysins are increasingly being genetically modified and produced recombinantly, which is relatively easy to achieve due to their modular structure consisting of lytic and binding domains. Endolysins have already found increasing actual or extensively postulated use for preventive, therapeutic, and diagnostic purposes in human and veterinary medicine as well as in food safety, biotechnology, and the One Health sector; however, this still needs to be better substantiated by valid studies. Although, in contrast to phage therapy, the regulatory aspects can follow the approval procedures also required for other pharmaceuticals, only less than a dozen randomized controlled studies of phases 1 to 3 have been initiated or completed in the field of human medicine. Only very few endolysin formulations approved as medical products are currently available on the market and approval as medicinal drugs is being sought for some endolysins.

## Einleitung

Antimikrobielle Substanzen mit enzymatischer Aktivität (sog. Enzybiotika [1]) stellen neben der Phagentherapie eine weitere Alternative zu den klassischen Antibiotika dar [2]. Neben Enzybiotika mit bakterieller Herkunft, wie z. B. das bakteriolytisch wirkende Lysostaphin von *Staphylococcus simulans*, haben in den letzten Jahren insbesondere Bakteriophagen-assoziierte Enzybiotika an Interesse gewonnen [3]. Zwei Gruppen sind hier zu unterscheiden, Endo- und Exolysine, die beide lytisch auf die bakterielle Zellwand wirken, wobei die Endolysine zum Zelltod führen. Während Endolysine gegen Ende des viralen Replikationszyklus gebildet werden und generalisiert lytisch von innen („lysis from within“) auf die Zellwand mit dem Ziel der Freisetzung der gebildeten viralen Partikel wirken, sind Exolysine (Ektolysine) viriongebunden und attackieren lokalisiert die Zellwand von außen („lysis from without“), um dem Phagen zu ermöglichen, sein genetisches Material in die Bakterienzelle zu injizieren [4]. Sie werden deshalb auch als Virion- oder „tail“-assoziierte bzw. strukturelle Lysine (VALs, „virion-associated lysins“, bzw. VAPGHs, „virion-associated peptidoglycan hydrolases“) bezeichnet (Details zu Wirkung, Einsatz und Studienlage, siehe [4]). Jedoch können auch Endolysine ihre enzymatische und damit lytische Wirkung auf die bakterielle Zellwand von außen entfalten, wenn sie entsprechend appliziert werden.

Ein erster Nachweis eines lytischen Faktors aus Phagenfiltraten gelang W. R. Maxted im Jahr 1957 [5], gefolgt von der Aufreinigung des C1-Lysins aus dem „Evans B563 (C1)-Phagen“ durch R. Krause [6]. Der Begriff „Endolysin“, der 1958 von Jacob und Fuerst für eine bakteriolytische Substanz vom λ‑Phagen geprägt wurde [7], wird heute für Enzyme verwendet, die die glykosidischen bzw. Peptidbindungen lösen, die dem Peptidoglykan der bakteriellen Zellwand die Stabilität verleihen [8]. Vor allem in älterer Literatur wurden Endolysine auch als Lysozyme (z. B. T7-Lysozym) bezeichnet. Erste therapeutische Anwendungen erfolgten nach der Jahrtausendwende insbesondere gegen grampositive Erreger, da bei diesen die Endolysinaktivität direkt wirksam werden kann. Bei gramnegativen Bakterien muss das Endolysin so modifiziert werden, dass es erst die für diesen Zellaufbau typische äußere Membran überwinden kann. Zu diesem Zweck sowie u. a. zur Optimierung der Endolysinspezifität und Verbesserung ihrer Stabilität werden Endolysine zunehmend gentechnisch modifiziert und rekombinant produziert. Ihre auch nach arzneimittel- bzw. medizinproduktrechtlichen Erfordernissen relativ einfache Herstellbarkeit und Modulierbarkeit führten rasch zu einem breiten Interesse an therapeutischen Einsatzmöglichkeiten in der Humanmedizin, die im Artikel vorrangig betrachtet werden sollen. Darüber hinaus sind vielversprechende Anwendungen in der Veterinärmedizin, Lebensmittelproduktion, Diagnostik und Biotechnologie bereits bekannt.

Im Gegensatz zur Therapie mit natürlichen bzw. gentechnisch veränderten Phagen lassen Endolysine eine Reihe von Vorteilen erwarten, wie eine universellere Anwendbarkeit über Stammgrenzen hinweg, unwahrscheinlichere Resistenzentwicklung, einfachere Modifizierbarkeit, unkompliziertere Formulierung, simplere Pharmakokinetik (Tab. 1) und nicht zuletzt weniger komplizierte regulatorische Aspekte (siehe Beitrag von T. Faltus und T. Buss et al. in diesem Themenheft). In der Literatur ist in den letzten Jahrzehnten eine Vielzahl von Endolysinen unterschiedlichster Phagenherkunft und gentechnischer Modifikation beschrieben worden [9–11], jedoch nur sehr wenige haben das Stadium der klinischen Erprobung erreicht, auf die sich der vorliegende Beitrag fokussiert.

**Tab. 1:** Aspekte zum therapeutischen Einsatz von Phagen-Endolysinen, intakten Phagen und Antibiotika im Vergleich

Aspekt	Phagen-Endolysine	Bakteriophagen	Antibiotika
*Beschreibung*	Natürlich vorkommende Enzyme der Bakteriophagen; gentechnisch modifizierbar	Natürlich vorkommende infektiöse Agenzien (Bakterien-befallende Viren), gentechnisch modifizierbar	Natürliche, semisynthetische oder synthetische Wirkstoffe mit antibakterieller Wirkung
*Wirkspektrum*	Sehr schmal (zumeist stamm- bzw. speziesspezifisch); gentechnisch zuschneidbar	Sehr schmal Phagotyp-spezifisch (häufig stamm- bzw. speziesspezifisch)	Breit bis sehr breit (je nach Wirkungsweise)
*Einfluss auf die Mikrobiota*	Sehr gering	Sehr gering	Hoch (je nach Wirkspektrum)
*Wirkprinzip*	Bakterizid (bakteriolytisch)	Bakterizid (bakteriolytisch)	Bakteriostatisch oder bakterizid
*Wirkung auf Biofilme*	Möglich; gentechnisch modifizierbar	Möglich; gentechnisch modifizierbar	Substanzabhängig
*Resistenzentwicklung*	Unwahrscheinlich	Schnell (innerhalb einer Applikation möglich)	Langsam bis schnell (substanzabhängig)
*Horizontaler Resistenzgentransfer*	Nicht möglich	Nicht beschrieben	Häufig (über mobile genetische Elemente)
*Vertikaler Resistenzgentransfer*	Nicht möglich	Regelmäßig	Regelmäßig
*Antibiotika-Kreuzresistenz*	Keine	Keine	Einzel‑, Multi- bis Panresistenz (je nach Resistenzmechanismus)
*Wirkungskinetik*	Sehr schnell (Minuten-Stunden)	Langsam (nach Etablierung des lytischen Zyklus; ggf. erst nach einigen Zyklen)	Schnell (Stunden; abhängig von bakterizider bzw. bakteriostatischer Wirkung)
*Pharmakokinetik*	Kontrollierte Dosierung mit kalkulierbarer Bioverfügbarkeit, Aktivität, Metabolisierung und Ausscheidung	Kontrollierte Dosierung, weiterer Verlauf (Selbstreplikation) abhängig vom Vorhandensein von Target-Organismen und deren Quantität und Verteilung	Kontrollierte Dosierung mit kalkulierbarer Bioverfügbarkeit, Aktivität, Metabolisierung und Ausscheidung
*Unerwünschte Wirkungen*	Unwahrscheinlich bei topischer Applikation; bei systemischer Applikation potenzielle Immunogenität	Relativ gering (bei lytischen Phagen), ansonsten potenzielle Gefahr durch von Prophagen kodierte Toxingene und Wirtszell-Beimengungen	Gering bis hoch (je nach Substanzeigenschaften)
*GMP-Herstellung*^*a*^	Unproblematisch biotechnologisch	Kaum umgesetzt (aufwendig)	Unproblematisch chemisch bzw. biotechnologisch
*Notwendigkeit der Produktaktualisierung*	Unwahrscheinlich	Häufig aufgrund von Resistenzentwicklung und Erreger(stamm)wechsel	Keine (allerdings Unwirksamkeit durch Resistenzentwicklung)
*Regulative Aspekte und Zulassung*	International etabliert (Arzneimittel oder Medizinprodukt)	In einigen Ländern/Regionen etabliert (auch als individueller Heilversuch), aktuelle Aufnahme in das Europäische Arzneibuch (Pharmacopoeia Europaea)	International etabliert (Arzneimittel)

^a^*GMP* Good Manufacturing Practice (Gute Herstellungspraxis)

## Struktur und Wirkmechanismus

### Natürliche Endolysine

**Abb. 1:**
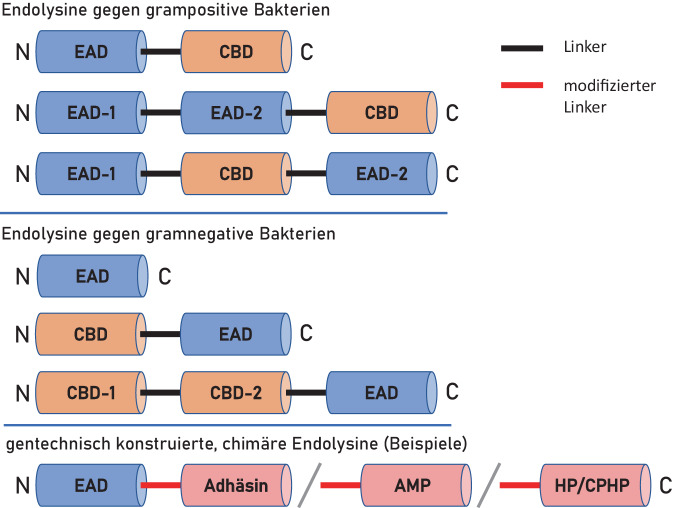
Schematische Darstellung natürlicher und chimärer Endolysine von Bakteriophagen mit Spezifität gegen grampositive oder gramnegative Bakterien bestehend jeweils aus enzymatisch/katalytisch aktiven Domänen („enzymatically active domain“ – *EAD*) und Zellwand-bindenden Domänen („cell wall-binding domain“ – *CBD*) bzw. weiteren, gentechnisch eingefügten Bestandteilen, die durch molekulare Linker verbunden sind. Chimäre Endolysine können u. a. mit Adhäsinen, antimikrobiellen Peptiden (*AMP*) oder „homing peptides“ (*HP*) bzw. „cell-penetrating homing peptides“ (*CPHP*) fusioniert sein

Endolysine degradieren überwiegend als Hydrolasen („phage-derived peptidoglycan hydrolases“ – PGH) die bakterielle Zellwand, deren Hauptkomponente Peptidoglykan (Murein) in Form eines dicken 3‑dimensionalen Netzes ist; hinzukommen bei grampositiven Bakterien „sekundäre Zellwandpolymere“ („secondary cell wall polymers“ – SCWPs). Weiterhin können auch lytische Phagen-Transglykosidasen das Peptidoglykan durch nichthydrolytische Mechanismen strukturell schwächen. Die Peptidoglykanstruktur ist innerhalb einer Gattung in hohem Maße konserviert, die genaue molekulare Beschaffenheit des Peptidoglykans und der SCWPs kann jedoch bis hinunter auf Spezies- und Stammebene spezifisch sein [12]. Somit entstand im Laufe der Bakterien-Phagen-Koevolution im Gegenzug eine recht große Vielfalt an Endolysinen, die sich in ihrer Struktur, katalytischen Aktivität, Enzymkinetik und insbesondere in ihrer Spezifität unterscheiden [13]. Endolysine von Phagen, die Bakterien mit grampositivem Zellwandaufbau (einschließlich Mykobakterien) befallen, unterscheiden sich strukturell von denjenigen, deren Phagen gramnegative Bakterien infizieren (Abb. 1; [8]).

Wie anhand der Aufklärung der Kristallstruktur bestätigt werden konnte, sind Endolysine modular aufgebaut [14]. Die Endolysine von Phagen mit grampositiver Wirtsspezifität besitzen zumeist ein Molekulargewicht von 15–40 kDa. Sie bestehen aus einer oder zwei enzymatisch aktiven Domänen („enzymatically active domains“ – EADs; auch „catalytic domain“ – CD), die N‑terminal bzw. zusätzlich zentral angeordnet sind, sowie einer C‑terminalen Zellwand-bindenden Domäne („cell wall-binding domain“ – CBD). Die Domänen sind durch einen kurzen, flexiblen Linker verbunden. Die EAD können Aktivitäten verschiedener Enzymklassen (Glykosidasen, Amidasen und Endopeptidasen) aufweisen und somit unterschiedliche Bindungen der Peptidoglykanstruktur der bakteriellen Zellwand attackieren. Beispielsweise handelt es sich bei der N‑terminalen EAD von Staphylokokken-Phagen in den meisten Fällen um eine cystein- und histidinabhängige Amidohydrolase/Peptidase (CHAP), die als D‑Ala-Gly-Endopeptidase die Bindung zwischen D‑Ala des Peptidstrangs und der Pentaglycinbrücke des Peptidoglykans spaltet. Bei Endolysinen mit mehr als zwei Domänen können auch Molekulargewichte von > 50 kDa erreicht werden. Sowohl die Architektur des Peptidoglykans anderer grampositiver Erreger als auch der Endolysine der diese Arten befallenden Phagen weisen hierzu Unterschiede auf. Die bis vor kurzem bekannten Endolysine der gramnegative Bakterien befallenden Phagen sind leichter (15–20 kDa), globulär strukturiert und sind mit einer alleinigen EAD simpler aufgebaut. Neue Studien zeigen, dass bei diesen Phagen auch eine modulare Endolysinkonfiguration vorkommen kann, die ein oder zwei CBDs am N‑Terminus aufweisen, während sich das EAD-Modul am C‑Terminus befindet, also eine im Vergleich zu Phagen mit grampositiver Spezifität inverse Ausrichtung der molekularen Struktur [15].

Die CBDs sind für die nichtkovalente Bindung an verschiedene Liganden der bakteriellen Zellwandoberflächen, wie z. B. Peptidoglykanbestandteile, Lipopolysaccharide, Teichonsäuren, Peptide oder Kohlenhydrate, verantwortlich und vermitteln die enzymatische Aktivität der EAD [16, 17]. Ihre Bindung ist wirtsspezifisch, von speziesübergreifend bis spezies- und stammspezifisch [13]. Die auf Basis umfangreicher Phagenbibliotheken früher übliche Methodik zur Stammtypisierung von Staphylokokken, Listerien und anderen Bakterien mittels Phagentypisierung (Phagotyping; [18]) beruht hingegen auf der spezifischen Bindung der Phagenrezeptorbindeproteine, z. B. der Phagenschwanzfasern, an bakterielle Wirtsoberflächenrezeptoren.

Bei den meisten Phagen werden für die natürliche Endolysinwirkung von innen zusätzlich noch kleine hydrophobe Proteine, sog. Holine, benötigt, um zuerst die Zytoplasmamembran perforieren zu können, bevor die Zellwand für die Endolysine erreichbar wird. Für die gramnegativen Bakterien wird noch eine andere Gruppe von Proteinen, die sog. Spanine, erforderlich, um auch die äußere Membran penetrieren zu können [19]. Diese stellt für natürliche Endolysine, wenn sie von außen wirken sollen, also z. B. im therapeutischen Einsatz, eine lyseverhindernde Barriere bei den gramnegativen Bakterien dar.

### Gentechnisch modifizierte Endolysine

Mit der Aufklärung der Endolysinstruktur und der Funktion der einzelnen Strukturkomponenten [14] sowie den gentechnischen Möglichkeiten zur Modifizierung der Struktur der natürlichen Endolysine eröffnete sich eine Reihe von Möglichkeiten, um Limitationen für den Einsatz von Endolysinen insbesondere im medizinischen Bereich zu überwinden. Hierzu zählen in erster Linie eine Ausdehnung der oft zu schmalen Target-Spezifität und die Anwendungsausweitung auf gramnegative Bakterien sowie Verbesserungen der Endolysinstabilität und -pharmakokinetik. Anstrengungen in dieser Richtung führten zur gentechnischen Konstruktion chimärer Endolysinstrukturen (Chimäre von altgriech. Χίμαιρα – Mischwesen der griechischen Mythologie; [20]), die auch als „Chimeolysine“ („chimeolysins“; [21]) bezeichnet werden. Diese sind dadurch charakterisiert, dass adhäsions- und lysevermittelnde Bestandteile verschiedener Speziesherkunft (z. B. Phagen- und bakterielle Anteile) eingesetzt werden, die durch einen Linker verbunden sind. Weitere Funktionalisierungen der Endolysine, wie die Fusion mit zellpenetrierenden Peptiden zur Eliminierung intrazellulärer Bakterien oder mit „homing peptides“ als zielerkennende kurze Peptide zur gerichteten Anreicherung in bestimmten Geweben (auch in Kombination als „cell-penetrating homing peptides“ – CPHP), erweitern die zukünftigen therapeutischen und/oder diagnostischen Einsatzmöglichkeiten von Endolysinen in vielfältigster Weise [22].

Für chimäre Endolysine ist bereits eine Vielzahl von Kombinationen mit unterschiedlichster Spezifität beschrieben worden. Eine der ersten Anwendungen basierte auf der synergistischen Wirkung von Lysostaphin und LysK [23]. Lysostaphin wirkt als Zink-Endopeptidase auf die Polyglycinvernetzungen in der Peptidoglykanschicht der Staphylokokkenzellwand. Es handelt sich um ein Bakteriocin, das von *S. simulans* gebildet wird und als sogenanntes Staphylococcin mit hoher Aktivität *Staphylococcus aureus* eliminiert, während die Wirkung auf koagulasenegative Staphylokokken deutlich geringer ausfällt. Beim LysK handelt es sich um ein hochwirksames Endolysin [24] vom Bakteriophagen K aus der *Myoviridae*-Familie, einem Phagen mit vergleichsweise breiter Wirtsaktivität gegen koagulasepositive und -negative Staphylokokken, wobei die CHAP-Domäne für die Wirkung essenziell ist [25]. Das rekombinante PRF-119 (Hyglos GmbH, Bernried, Deutschland) und dessen stabilitätsverbesserte Weiterentwicklung HY-133 (Hyglos, später HYPharm GmbH, Bernried, Deutschland) wurden als erste Lysine nach diesem Prinzip als Chimäre aus der CHAP-Domäne vom Bakteriophagen K (CHAP_K_) und der CBD von Lysostaphin generiert und umfangreich auf deren antibakterielle Aktivität getestet [26, 27]. PRF-119/HY-133 zeigten sich hoch aktiv gegenüber mehr als 1000 *S.-aureus*-Isolaten, einschließlich Hospital‑, Community- und Livestock-associated MRSA (Methicillin-resistenter *S. aureus*) der verschiedensten klonalen Linien, weiteren Resistenzphänotypen, einschließlich Mupirocinresistenz und Small-Colony-Variant-(SCV-)Phänotypen, die intrazellulär persistierende Varianten darstellen [26–28]. Untersuchungen zur Bakterizidie ergaben Werte der minimalen bakteriziden Konzentrationen (MBK) in der Größenordnung der minimalen Hemmkonzentrationen (MHK; MHK_50/90_ und MBK_50/90_ jeweils im Bereich von 0,12–0,5 mg/L) bei sehr schnellem, konzentrationsabhängigem Wirkungseintritt innerhalb von wenigen Minuten bis ca. 2 h [29].

Das Prinzip der Ergänzung von CHAP_K_ oder anderer katalytischer CHAP-Domänen (z. B. LysRODI und LysC1C) um die Lysostaphin-CBD oder andere Domänen (z. B. M23-Endopeptidase oder SH3b) wurde ebenfalls von anderen Arbeitsgruppen u. a. zur Aktivitäts- und Spezifitätsverbesserung für Staphylokokken-wirksame Endolysine eingesetzt [30–33]. Auch gegen andere bakterielle Pathogene wurde bereits eine Reihe chimärer Lysine konstruiert. Mit ClyR konnte ein Chimeolysin mit sehr guter Aktivität und insbesondere einem erweiterten Wirtsspektrum gegen die meisten Streptokokkenarten (u. a. *S. pyogenes, S. agalactiae, S. dysgalactiae, S. mutans, S. pneumoniae*) sowie einige Enterokokken- und Staphylokokkenarten konstruiert werden [34]. Obwohl bisher keine *Gardnerella*-spezifischen Bakteriophagen bekannt sind, konnten DNA-Regionen in den Genomen identifiziert werden, die auf einen Prophagenursprung hindeuten [35]. Endolysin-typische EAD- und CBD-Sequenzen wurden als rekombinante Proteine exprimiert und die Kombination mit der höchsten Bakterizidie (PM-477) wurde weiter untersucht und zeigte eine hohe Aktivität gegen *G. vaginalis,* selbst in Biofilmen [36].

Eine Möglichkeit zur Überwindung der Gramnegativenproblematik ist die Fusion eines Endolysins mit Molekülen, die in der Lage sind, die äußere Membran zu permeabilisieren. Ein Beispiel ist das gentechnisch konstruierte Hybridtoxin aus der FyuA-(„ferric-yersiniabactin-uptake“-)Transporter-Bindungsdomäne von Pesticin und dem N‑Terminus vom T4-Lysozym [37]. Das von *Yersinia pestis* produzierte Pesticin wird unter Stressbedingungen als Bakterizidin produziert, um verwandte Stämme zu eliminieren. Erst die Wechselwirkung zwischen dem Outer-Membrane-Protein FyuA und Pesticin ermöglicht den Import von Pesticin durch die äußere Membran in das Periplasma. Dort entfaltet es seine bakterizide Wirkung, indem es das Peptidoglykan durch seine Muramidase-(Lysozym‑)Aktivität abbaut. Pesticin-exprimierende Stämme schützen sich allerdings selbst, indem sie ein Immunitätsprotein („pesticin immunity protein“ – Pim) exprimieren, das die Aktivität des Toxins hemmt. Das Hybridtoxin konnte spezifisch *Yersinia*- und pathogene *Escherichia-coli*-Stämme lysieren und außerdem die Pim-Wirkung umgehen, was ihm einen deutlichen Vorteil gegenüber dem alleinigen Pesticineinsatz verschafft [37]. Die Aktivität konnte durch einen verlängerten Linker zwischen dem Peptid und dem Endolysin oder durch eine Kombination von polykationischen und hydrophoben/amphipathischen Peptiden weiter gesteigert werden [38]. Für derartige Fusionsproteine aus Endolysinen und antimikrobiellen Peptiden mit membranpenetrierenden Eigenschaften wurde die Bezeichnung „Artilysine“ eingeführt [38].

Interessant sind Fusionen mit Cecropinen, die als antimikrobielle Peptide zum innaten Immunsystem von Insekten gehören und durch ihre amphipathische Natur die Permeabilisierung der äußeren Membran erleichtern. Das Artilysin eLysMK34 als Fusionsprodukt aus dem *Acinetobacter-baumannii*-Phagen „MK34-Lysin“ und Cecropin A zeigte u. a. eine verbesserte Abtötungskinetik im Vergleich zum Wildtyplysin [39]. Weitere Möglichkeiten zur Überwindung der äußeren Membran sind Endolysinfusionen mit rezeptorbindenden Phagenproteinen (Innolysine; [40]) und Pyocindomänen (Lysocine; [41]).

## Einsatzmöglichkeiten und deren Wertung

Ihre relativ einfache gentechnische Modifizierbarkeit macht Endolysine zu aussichtsreichen Kandidaten für die unterschiedlichsten Anwendungen nicht nur zu therapeutischen, kosmetischen und diagnostischen Zwecken in der Human- und Veterinärmedizin, sondern auch in der Lebensmittelindustrie für Biokonservierung und Lebensmittelsicherheit, in der Landwirtschaft und Umwelt zur biologischen Schädlingsbekämpfung und Bioremediation sowie zu vielfältigen Zwecken in der Biotechnologie.

### Präventive und therapeutische Anwendungen

Die im Gegensatz zu den klassischen Antibiotika quasi phylogenetisch ableitbare und damit klar definierbare Spezifität auf Genus‑, Spezies- oder Stammebene ertüchtigt Endolysine zu idealen Wirkstoffen im Sinne des Konzepts einer ultimativ schmalen, nur auf das kausale Agens ausgerichteten Therapie bakterieller Infektionen, im Idealfall ohne jegliche Beeinträchtigung der Mikrobiota des Wirtes. Dieses Prinzip einer möglichst weitgehenden Vermeidung von Antibiotikaresistenzselektionsdrücken ist von noch größerer Bedeutung im präventiven Bereich, z. B. zur Vermeidung postoperativer Wundinfektionen. Auch aus dieser Sicht sind Endolysine vielversprechende Präparate, wie u. a. zur Eradizierung einer nasalen MRSA/*S.-aureus*-Kolonisation als Quelle nachfolgender nosokomialer Infektionen [42].

Perfekt ergänzend zur bakteriziden Wirkung ist die Fähigkeit einiger Endolysine, mit bakteriellen Biofilmen von grampositiven und gramnegativen Erregern zu interagieren und diese abzubauen [43]. Es konnte z. B. für Biofilme von *Klebsiella pneumoniae* und *Acinetobacter baumannii* gezeigt werden, dass enzymatische Endolysinaktivität mit Biofilm-protektiven Verbindungen wie extrazellulärer DNA und polyanionischen Kohlenhydraten interagiert und Biofilm-zerstörende Effekte auslöst [44]. Für detaillierte Auflistungen der Vielzahl bereits beschriebener Endolysine und anderer Phagenproteine für einen potenziellen präventiven und/oder therapeutischen Einsatz, die sich noch im experimentellen oder frühen klinischen Stadium befinden, sei hier auf aktuelle Übersichtsarbeiten verwiesen [45–50].

Da der lytische Wirkmechanismus von Endolysinen unabhängig von bakteriellen Resistenzmechanismen gegen Antibiotika ist, sind Kreuzresistenzen mit Antibiotika ohne Relevanz. Das Potenzial von Resistenzentwicklungen gegen eingesetzte Endolysine ist gleichfalls niedrig. Hinzu kommt die ultraschnelle bakterizide Wirkung von Endolysinen innerhalb weniger Minuten bis Stunden, die den Zeitraum für einen hypothetischen Selektionsdruck extrem kurz hält [29]. Der schnelle Wirkungseintritt ist von besonderem Interesse für präventive Zwecke, da z. B. präoperative Wartezeiten bis zu einer erfolgreichen MRE-Dekolonisierung von derzeit mehreren Tagen drastisch reduziert werden könnten. Topisch eingesetzt lässt sich ihre Aktivität ohne systemische Wirkung streng lokal begrenzen.

Phänotypische Varianten mit reduziertem Metabolismus, oft in Kombination mit intrazellulärer Persistenz oder Biofilmeinbettung, sind eine der Hauptursachen für eine funktionelle Resistenz gegenüber *in vitro* als empfindlich getesteten Antibiotika und können die Ursache für chronisch persistierende und rezidivierende Infektionsverläufe bilden. Derartige Persister‑, Dormanz- oder Small-Colony-Variant-(SCV-)Stadien können durch Phagenendolysine erfolgreich lysiert werden [28, 51]. Im Gegensatz zum Einsatz von Phagenendolysinen ist für die Therapie mit kompletten Phagen bekannt, dass diese die Entstehung von Persisterzellen sogar induzieren können [52, 53].

Weitere vorteilhafte Faktoren umfassen die geringe Wahrscheinlichkeit von Nebenwirkungen sowohl bei topischer als auch parenteraler Applikation aufgrund ihrer sehr geringen Toxizität [54, 55]. Translations- und anwendungsrelevante Vorteile wie die hohe Vielfalt natürlich vorkommender Endolysine und deren unkomplizierte gentechnische Modifizierbarkeit, die einfache Produktion, einschließlich unter Bedingungen der Guten Herstellungspraxis (Good Manufacturing Practice – GMP), ihre gute Stabilität (ggf. nach gentechnischer Optimierung), die vielfältigen Formulierungsmöglichkeiten (z. B. als Lösungen, Salben oder Sprays) runden das Bild einer potenziell erfolgreichen, neuen Antiinfektivasubstanzklasse ab.

Bei Proteinbiopharmazeutika besteht grundsätzlich die Möglichkeit einer unerwünschten Auslösung von Immunreaktionen, die bei parenteraler Applikation über die Generierung neutralisierender Antikörper unter Umständen zu einem Verlust der Wirksamkeit des Medikaments sowie zur Autoimmunität und zu Überempfindlichkeitsreaktionen führen können. In einer Studie zur möglichen Endolysininaktivierung durch Antikörper konnte gezeigt werden, dass LysGH15-spezifische Antikörper die Abtötungseffizienz des Endolysins gegen MRSA weder *in vitro* noch im Mausexperiment *in vivo* beeinträchtigten [56].

### Endolysine in klinischer Erprobung

Im Gegensatz zu der großen Zahl bekannter und charakterisierter Endolysinmoleküle befinden sich nur sehr wenige davon in der klinischen Entwicklung bzw. wurden oder werden in klinischen Studien an Menschen untersucht. Eine Übersicht von bisher stattgefundenen und laufenden kontrollierten klinischen Studien mit Endolysinen ist in der Tab. 2 zusammengefasst. Bisher wurden nur gegen *S. aureus* gerichtete Endolysine in klinischen Studien untersucht, wobei zwischen zwei Indikationen bzw. Verabreichungswegen unterschieden werden kann. Während einige Substanzen für eine topische Anwendung entwickelt werden, zielen die anderen auf eine intravenöse Verabreichung ab. Interessant könnte auch eine Kombinationstherapie von Endolysinen und klassischen Antibiotika sein [57].

**Tab. 2:** Übersicht über randomisierte kontrollierte klinische Studien (*RCT*) mit Endolysinen

Prüfpräparat	Studiendesign	Zielorganismus	Verwendungszweck	Verabreichungsweg	Registrierung^a^	Literatur
AP-SA02	RCT, verblindet, Phase I/II	*S. aureus*	Bakteriämie	Intravenös	NCT04160468	n. a.
Exebacase (CF-301)	RCT, verblindet, Phase III	*S. aureus*	Endokarditis, Bakteriämie	Intravenös	NCT04160468	Fowler, Jr. et al. (2024; [58])
Exebacase (CF-301)	RCT, verblindet, Phase II	*S. aureus*	Endokarditis, Bakteriämie	Intravenös	NCT03163446	Fowler, Jr. et al. (2020; [57])
Exebacase (CF-301)	RCT, verblindet, Phase I	*S. aureus*	Endokarditis, Bakteriämie	Intravenös	NCT02439359	n. a.
HY-133	RCT, verblindet, Phase I/II	*S. aureus*	Eradikation der nasalen Kolonisation	Intranasal	NCT06290557	n. a.
LSVT-1701 (SAL200)	RCT, verblindet, Phase I	*S. aureus*	Bakteriämie	Intravenös	NCT03446053	Wire et al. (2022; [73])
N‑Rephasin (SAL200)	RCT, verblindet, Phase II	*S. aureus*	Bakteriämie	Intravenös	NCT03089697	n. a.
N‑Rephasin (SAL200)	RCT, verblindet, Phase I	*S. aureus*	Bakteriämie	Intravenös	NCT01855048	Jun et al. (2017; [74])
P128	RCT, verblindet, Phase I/II	*S. aureus*	Nasale Eradikation	Intranasal	NCT01746654	n. a.
Staphefekt (SA100)	RCT, verblindet	*S. aureus*	Atopische Dermatitis	Hautcreme	NCT02840955	Totté et al. (2017; [65]), de Wit et al. (2019; [75])

*RCT* randomisierte kontrollierte Studie („randomized controlled trial“), *n.* *a.* nicht verfügbar („not available“)

^a^National Clinical Trials (NCT) Identifier Number, Identifikationsnummer der klinischen Studie in der Datenbank „ClinicalTrials.gov“ der U.S. National Library of Medicine

Unter den topischen Endolysinen in der klinischen Entwicklung ist das rekombinant hergestellte, chimäre HY-133 zu erwähnen, welches gerade in einer deutschen klinischen Phase-I/II-Studie insbesondere auf seine Sicherheit und Verträglichkeit, aber auch auf Wirksamkeit zur nasalen Eradikation von *S. aureus* untersucht wird. Diese doppelblinde randomisierte kontrollierte Studie (NCT06290557) befindet sich aktuell (Stand März 2025) in der aktiven Phase der Probandenrekrutierung. Neben einer einzelnen Verabreichung des Wirkstoffs sollen nach einer Interimssicherheitsbewertung multiple Applikationen sowie die Verabreichung der erhöhten Dosierungen des Wirkstoffes erfolgen.

Unter den Endolysinen für eine systemische Anwendung hat der Wirkstoff Exebacase (CF-301), der zur intravenösen Therapie von der durch *S. aureus* verursachten Endokarditis bzw. Bakteriämie entwickelt wird, die meisten klinischen Studien absolviert. Nach einer Phase-I-Studie (NCT02439359), in der die Sicherheit von Exebacase an gesunden Probanden gezeigt wurde, folgten die US-amerikanischen Studien der Phase II und Phase III. Die Ergebnisse der Phase-II-Studie im Design einer randomisierten, Placebo-kontrollierten Überlegenheitsstudie waren vielversprechend. Die klinische Ansprechrate betrug am Tag 14 in der Gruppe, die Exebacase zusammen mit einem Standardantibiotikum erhielt, 70,4 %, während die Ansprechrate in der Gruppe, die nur Antibiotika appliziert bekam, bei 60,0 % lag (*p* = 0,31). Signifikant war der Effekt von Exebacase in der MRSA-Subgruppe (74,1 % vs. 31,3 %, *p* = 0,01; [57]). Allerdings konnte in der Phase-III-Studie (DISRUPT), die gleichfalls als randomisierte Überlegenheitsstudie angelegt worden war, kein positiver Effekt der zusätzlichen Gabe von Exebacase bestätigt werden [58]. Die klinischen Ansprechraten am Tag 14 betrugen in der MRSA-Population 50,0 % bei kombinierter Gabe von Exebacase plus Antibiotikum und 60,6 % bei alleiniger Verabreichung von Antibiotika (*p* = 0,392). Angesichts der vielversprechenden Ergebnisse der Phase-II-Studie war dieses Ergebnis unerwartet und die Studie musste nach der Zwischenbewertung des Datenüberwachungskomitees vorzeitig abgebrochen werden [58]. Von den Autoren werden eine Heterogenität innerhalb der Studienpopulation und ein relativ geringer Stichprobenumfang diskutiert. Weitere Endolysine befinden sich zurzeit in der Entwicklungs-Pipeline für unterschiedliche klinische Indikationen (Tab. 2).

Zu erwähnen sind neben den in Tab. 2 aufgeführten kontrollierten Endolysinstudien eine nichtkontrollierte klinische Studie an Patienten mit atopischer Dermatitis, die mit Staphefekt™ SA.100 behandelt wurden [59], sowie Fallberichte zur Behandlung von Hauterkrankungen, die mit *S. aureus* assoziiert werden [60], und Knieprotheseninfektionen durch *S. epidermidis* [61].

Marktverfügbar sind bereits Medizinprodukte als rezeptfrei erhältliche Hautcremes oder Gele mit gentechnisch modifizierten Endolysinen als Inhaltsstoff, die als Mikrobiom-freundlich/-unterstützend beworben werden. Das betrifft Staphefekt™ SA.100 (auch als Endobioma™ vermarktet), das zur topischen Behandlung oder deren Unterstützung bei atopischem Ekzem/Neurodermatitis und Rosacea angeboten wird. Die Studienlage zu diesen Produkten ist sehr begrenzt und die Zulassung von Phagenendolysinen als Medizinprodukt anstatt als Arzneimittel ist zu hinterfragen.

### Weitere Endolysinanwendungen

Neben einem therapeutischen bzw. präventiven Einsatz der Endolysine in der Medizin ist eine Reihe weiterer Einsatzgebiete bereits beschrieben. Ein vielversprechender Bereich ist das sog. Mikrobiom-Engineering. Obwohl die Bedeutung des Mikrobioms für die menschliche Gesundheit zunehmend erkannt und berücksichtigt wird, fehlt es bisher an einer breiten Palette maßgeschneiderter Werkzeuge, um gezielt die Zusammensetzung der Mikrobiota zu modulieren, insbesondere wenn es gilt, einzelne Stämme oder Spezies ohne „Kollateralschaden“ für andere Mikroorganismen zu eradizieren. Gerade hier können Endolysine im Gegensatz zu den Antibiotika ihre Vorteile bezüglich Spezifität und relativ einfacher gentechnischer Modifizierbarkeit zum Tragen bringen und machen sich so zu aussichtsreichen Kandidaten für diesen Zweck [62]. Erste Ergebnisse aus In-vitro‑, Ex-vivo- und In-vivo-Studien untermauern mögliche Einsatzgebiete, wie u. a. die Therapie einer Infektion mit *Clostridioides (Clostridium) difficile* im Rahmen einer Antibiotika-assoziierten Durchfallerkrankung zur Remodulierung der Darmmikrobiota [63], die präoperative Eradizierung einer *S.-aureus*/MRSA-Besiedlung der Nasenhöhle bzw. von geschädigter Haut [64–66] oder die spezifische Beseitigung von *Gardnerella vaginalis* aus der Vaginalmikrobiota bei bakterieller Vaginose [36].

Die gleichen Vorteile einer hohen Spezifität und gentechnischen Modifizierbarkeit lassen Endolysine auch als ideale Biosonden zum Einsatz in der mikrobiologischen Diagnostik erwarten. Neben CBDs von Endolysinen stellen rezeptorbindende Phagenproteine („tail fibers“ und „tailspikes“) einen Schlüssel für die Entwicklung von diagnostischen Werkzeugen u. a. für die Markierung, die Immobilisierung und den Nachweis definierter bakterieller Targets dar [40]. Hierzu können – als Ersatz von diagnostisch eingesetzten Antikörpern – fluoreszenz- oder enzymmarkierte CBDs bzw. rezeptorbindende Phagenproteine z. B. zur immunomagnetischen Separation von Pathogenen eingesetzt werden. Hauptsächlich für den Einsatz in der Lebensmittelproduktion ist bereits eine Reihe von Testsystemen mit Wildtyp- oder modifizierten Endolysinen beschrieben, u. a. zum Nachweis von *S. aureus* [67], Listerien [68], *Salmonella* Typhimurium [69] und *Clostridium perfringens* [70].

In der Lebensmitteltechnologie werden Endolysine bereits erfolgreich als Mittel zur unterstützenden Biokonservierung von Lebensmitteln eingesetzt [71]. Insbesondere thermophile Endolysine (> 65 °C) können hier in Zukunft breite Verwendung finden [72]. Für Anwendungsmöglichkeiten im One-Health-Bereich sei auf die Literatur verwiesen [72].

## Fazit

Die Identifizierung von neuen Phagenendolysinen und deren Charakterisierung mit dem Ziel eines möglichen Einsatzes in der Human- und Veterinärmedizin zu diagnostischen, präventiven und therapeutischen Zwecken sowie in vielen anderen Bereichen, wie der Lebensmitteltechnologie oder unter One-Health-Gesichtspunkten, stellen ein rasant wachsendes Forschungsfeld dar. Ihre natürliche Wirkungsbeschränkung auf definierte Taxa, ihre ultraschnelle Wirksamkeit und ihre relativ einfache gentechnische Modifizierbarkeit machen ihren Einsatz als zukünftige, maßgeschneiderte Produkte für die hochspezifische, Kollateralschäden-vermeidende Bekämpfung bakterieller Infektionen vielversprechend. Klinische Studien müssen jedoch noch zeigen, dass der in anderen Bereichen (z. B. Diagnostik und Lebensmitteltechnologie) bereits gelungene Einsatz rekombinanter Endolysine auch als Arzneimittel oder Medizinprodukt für die Human- und Veterinärmedizin umsetzbar ist.

## References

[1] Nelson D, Loomis L, Fischetti VA (2001) Prevention and elimination of upper respiratory colonization of mice by group A streptococci by using a bacteriophage lytic enzyme. Proc Natl Acad Sci U S A 98:4107–4112. 10.1073/pnas.061038398 11259652 10.1073/pnas.061038398 PMC31187

[2] Dams D, Briers Y (2019) Enzybiotics: Enzyme-Based Antibacterials as Therapeutics. Adv Exp Med Biol 1148:233–253. 10.1007/978-981-13-7709-9_11 31482502 10.1007/978-981-13-7709-9_11

[3] Borysowski J, Weber-Dąbrowska B, Górski A (2006) Bacteriophage endolysins as a novel class of antibacterial agents. Exp Biol Med 231:366–377. 10.1177/153537020623100402 10.1177/153537020623100402 16565432

[4] Rodríguez-Rubio L, Martínez B, Donovan DM, Rodríguez A, García P (2013) Bacteriophage virion-associated peptidoglycan hydrolases: potential new enzybiotics. Crit Rev Microbiol 39:427–434. 10.3109/1040841X.2012.723675 22991936 10.3109/1040841X.2012.723675

[5] Maxted WR (1957) The active agent in nascent phage lysis of streptococci. J Gen Microbiol 16:584–595. 10.1099/00221287-16-3-584 13439143 10.1099/00221287-16-3-584

[6] Krause RM (1957) Studies on bacteriophages of hemolytic streptococci. I. Factors influencing the interaction of phage and susceptible host cell. J Exp Med 106:365–384. 10.1084/jem.106.3.365 13463248 10.1084/jem.106.3.365 PMC2136776

[7] Jacob F, Fuerst CR (1958) The mechanism of lysis by phage studied with defective lysogenic bacteria. J Gen Microbiol 18:518–526. 10.1099/00221287-18-2-518 13525668 10.1099/00221287-18-2-518

[8] Young R (1992) Bacteriophage lysis: mechanism and regulation. Microbiol Rev 56:430–481. 10.1128/mr.56.3.430-481.1992 1406491 10.1128/mr.56.3.430-481.1992 PMC372879

[9] Hassannia M, Naderifar M, Salamy S, Akbarizadeh MR, Mohebi S, Moghadam MT (2023) Engineered phage enzymes against drug-resistant pathogens: a review on advances and applications. Bioprocess Biosyst Eng. 10.1007/s00449-023-02938-610.1007/s00449-023-02938-6 37962644 10.1007/s00449-023-02938-6

[10] Fenton M, Ross P, McAuliffe O, O’Mahony J, Coffey A (2010) Recombinant bacteriophage lysins as antibacterials. Bioeng Bugs 1:9–16. 10.4161/bbug.1.1.9818 21327123 10.4161/bbug.1.1.9818 PMC3035150

[11] Schmelcher M, Donovan DM, Loessner MJ (2012) Bacteriophage endolysins as novel antimicrobials. Future Microbiol 7:1147–1171. 10.2217/fmb.12.97 23030422 10.2217/fmb.12.97 PMC3563964

[12] Schleifer KH, Kandler O (1972) Peptidoglycan types of bacterial cell walls and their taxonomic implications. Bacteriol Rev 36:407–477. 10.1128/br.36.4.407-477.1972 4568761 10.1128/br.36.4.407-477.1972 PMC408328

[13] Catalão MJ, Gil F, Moniz-Pereira J, São-José C, Pimentel M (2013) Diversity in bacterial lysis systems: bacteriophages show the way. FEMS Microbiol Rev 37:554–571. 10.1111/1574-6976.12006 23043507 10.1111/1574-6976.12006

[14] Korndörfer IP, Danzer J, Schmelcher M, Zimmer M, Skerra A, Loessner MJ (2006) The crystal structure of the bacteriophage PSA endolysin reveals a unique fold responsible for specific recognition of Listeria cell walls. J Mol Biol 364:678–689. 10.1016/j.jmb.2006.08.069 17010991 10.1016/j.jmb.2006.08.069

[15] Briers Y, Volckaert G, Cornelissen A et al (2007) Muralytic activity and modular structure of the endolysins of Pseudomonas aeruginosa bacteriophages φKZ and EL. Mol Microbiol 65:1334–1344. 10.1111/j.1365-2958.2007.05870.x 17697255 10.1111/j.1365-2958.2007.05870.x

[16] Loessner MJ, Kramer K, Ebel F, Scherer S (2002) C‑terminal domains of Listeria monocytogenes bacteriophage murein hydrolases determine specific recognition and high-affinity binding to bacterial cell wall carbohydrates. Mol Microbiol 44:335–349. 10.1046/j.1365-2958.2002.02889.x 11972774 10.1046/j.1365-2958.2002.02889.x

[17] Hermoso JA, Monterroso B, Albert A et al (2003) Structural basis for selective recognition of pneumococcal cell wall by modular endolysin from phage Cp‑1. Structure 11:1239–1249. 10.1016/j.str.2003.09.005 14527392 10.1016/j.str.2003.09.005

[18] Blair JE, Williams RE (1961) Phage typing of staphylococci. Bull World Health Organ 24:771–784 (https://www.ncbi.nlm.nih.gov/pubmed/20604092) 20604092 PMC2555522

[19] Young R (2013) Phage lysis: do we have the hole story yet? Curr Opin Microbiol 16:790–797. 10.1016/j.mib.2013.08.008 24113139 10.1016/j.mib.2013.08.008 PMC3848059

[20] Manoharadas S, Witte A, Bläsi U (2009) Antimicrobial activity of a chimeric enzybiotic towards Staphylococcus aureus. J Biotechnol 139:118–123. 10.1016/j.jbiotec.2008.09.003 18940209 10.1016/j.jbiotec.2008.09.003

[21] Yang H, Bi Y, Shang X et al (2016) Antibiofilm Activities of a Novel Chimeolysin against Streptococcus mutans under Physiological and Cariogenic Conditions. Antimicrob Agents Chemother 60:7436–7443. 10.1128/AAC.01872-16 27736755 10.1128/AAC.01872-16 PMC5119027

[22] Wang Z, Kong L, Liu Y et al (2018) A Phage Lysin Fused to a Cell-Penetrating Peptide Kills Intracellular Methicillin-Resistant Staphylococcus aureus in Keratinocytes and Has Potential as a Treatment for Skin Infections in Mice. Appl Environ Microbiol. 10.1128/AEM.00380-18 29625989 10.1128/AEM.00380-18 PMC5981068

[23] Becker SC, Foster-Frey J, Donovan DM (2008) The phage K lytic enzyme LysK and lysostaphin act synergistically to kill MRSA. FEMS Microbiol Lett 287:185–191. 10.1111/j.1574-6968.2008.01308.x 18721148 10.1111/j.1574-6968.2008.01308.x

[24] Schmelcher M, Shen Y, Nelson DC et al (2015) Evolutionarily distinct bacteriophage endolysins featuring conserved peptidoglycan cleavage sites protect mice from MRSA infection. J Antimicrob Chemother 70:1453–1465. 10.1093/jac/dku552 25630640 10.1093/jac/dku552 PMC4398471

[25] Rountree PM (1949) The serological differentiation of staphylococcal bacteriophages. J Gen Microbiol 3:164–173. 10.1099/00221287-3-2-164 18144968 10.1099/00221287-3-2-164

[26] Idelevich EA, von Eiff C, Friedrich AW et al (2011) In vitro activity against Staphylococcus aureus of a novel antimicrobial agent, PRF-119, a recombinant chimeric bacteriophage endolysin. Antimicrob Agents Chemother 55:4416–4419. 10.1128/AAC.00217-11 21746950 10.1128/AAC.00217-11 PMC3165309

[27] Idelevich EA, Schaumburg F, Knaack D et al (2016) The recombinant bacteriophage endolysin HY-133 exhibits in vitro activity against different African clonal lineages of the Staphylococcus aureus complex, including Staphylococcus schweitzeri. Antimicrob Agents Chemother 60:2551–2553. 10.1128/AAC.02859-15 26833148 10.1128/AAC.02859-15 PMC4808236

[28] Schleimer N, Kaspar U, Knaack D et al (2019) In vitro activity of the bacteriophage endolysin HY-133 against Staphylococcus aureus small-colony variants and their corresponding wild types. Int J Mol Sci 20:E716. 10.3390/ijms20030716 10.3390/ijms20030716 PMC6387228 30736446

[29] Knaack D, Idelevich EA, Schleimer N et al (2019) Bactericidal activity of bacteriophage endolysin HY-133 against Staphylococcus aureus in comparison to other antibiotics as determined by minimum bactericidal concentrations and time-kill analysis. Diagn Microbiol Infect Dis 93:362–368. 10.1016/j.diagmicrobio.2018.11.005 30554844 10.1016/j.diagmicrobio.2018.11.005

[30] Gutiérrez D, Rodríguez-Rubio L, Ruas-Madiedo P et al (2021) Design and Selection of Engineered Lytic Proteins With Staphylococcus aureus Decolonizing Activity. Front Microbiol 12:723834. 10.3389/fmicb.2021.723834 34594314 10.3389/fmicb.2021.723834 PMC8477017

[31] Eichenseher F, Herpers BL, Badoux P et al (2022) Linker-Improved Chimeric Endolysin Selectively Kills Staphylococcus aureus In Vitro, on Reconstituted Human Epidermis, and in a Murine Model of Skin Infection. Antimicrob Agents Chemother 66:e227321. 10.1128/aac.02273-21 35416713 10.1128/aac.02273-21 PMC9112974

[32] Roehrig C, Huemer M, Lorge D et al (2024) MEndoB, a chimeric lysin featuring a novel domain architecture and superior activity for the treatment of staphylococcal infections. mBio. 10.1128/mbio.02540-23:e0254023 38275913 10.1128/mbio.02540-23 PMC10865858

[33] Schuch R, Lee HM, Schneider BC et al (2014) Combination therapy with lysin CF-301 and antibiotic is superior to antibiotic alone for treating methicillin-resistant Staphylococcus aureus-induced murine bacteremia. J Infect Dis 209:1469–1478. 10.1093/infdis/jit637 24286983 10.1093/infdis/jit637 PMC3982849

[34] Yang H, Linden SB, Wang J, Yu J, Nelson DC, Wei H (2015) A chimeolysin with extended-spectrum streptococcal host range found by an induced lysis-based rapid screening method. Sci Rep 5:17257. 10.1038/srep17257 26607832 10.1038/srep17257 PMC4660466

[35] Malki K, Shapiro JW, Price TK et al (2016) Genomes of Gardnerella Strains Reveal an Abundance of Prophages within the Bladder Microbiome. PLoS ONE 11:e166757. 10.1371/journal.pone.0166757 27861551 10.1371/journal.pone.0166757 PMC5115800

[36] Landlinger C, Tisakova L, Oberbauer V et al (2021) Engineered Phage Endolysin Eliminates Gardnerella Biofilm without Damaging Beneficial Bacteria in Bacterial Vaginosis Ex Vivo. Pathogens. 10.3390/pathogens10010054 33435575 10.3390/pathogens10010054 PMC7830407

[37] Lukacik P, Barnard TJ, Keller PW et al (2012) Structural engineering of a phage lysin that targets gram-negative pathogens. Proc Natl Acad Sci U S A 109:9857–9862. 10.1073/pnas.1203472109 22679291 10.1073/pnas.1203472109 PMC3382549

[38] Briers Y, Walmagh M, Van Puyenbroeck V et al (2014) Engineered endolysin-based “Artilysins” to combat multidrug-resistant gram-negative pathogens. mBio 5:e1379–1314. 10.1128/mBio.01379-14 24987094 10.1128/mBio.01379-14 PMC4161244

[39] Abdelkader K, Gutiérrez D, Tamés-Caunedo H et al (2022) Engineering a Lysin with Intrinsic Antibacterial Activity (LysMK34) by Cecropin A Fusion Enhances Its Antibacterial Properties against Acinetobacter baumannii. Appl Environ Microbiol 88:e151521. 10.1128/AEM.01515-21 34669452 10.1128/AEM.01515-21 PMC8752152

[40] Klumpp J, Dunne M, Loessner MJ (2023) A perfect fit: Bacteriophage receptor-binding proteins for diagnostic and therapeutic applications. Curr Opin Microbiol 71:102240. 10.1016/j.mib.2022.102240 36446275 10.1016/j.mib.2022.102240

[41] Heselpoth RD, Euler CW, Schuch R, Fischetti VA (2019) Lysocins: Bioengineered Antimicrobials That Deliver Lysins across the Outer Membrane of Gram-Negative Bacteria. Antimicrob Agents Chemother. 10.1128/AAC.00342-19 30962344 10.1128/AAC.00342-19 PMC6535517

[42] von Eiff C, Becker K, Machka K, Stammer H, Peters G (2001) Nasal carriage as a source of Staphylococcus aureus bacteremia. N Engl J Med 344:11–16. 10.1056/NEJM200101043440102 11136954 10.1056/NEJM200101043440102

[43] Golosova NN, Matveev AL, Tikunova NV et al (2024) Bacteriophage vB_SepP_134 and Endolysin LysSte_134_1 as Potential Staphylococcus-Biofilm-Removing Biological Agents. Viruses. 10.3390/v16030385 38543751 10.3390/v16030385 PMC10975630

[44] Lendel AM, Antonova NP, Grigoriev IV, Usachev EV, Gushchin VA, Vasina DV (2024) Biofilm-disrupting effects of phage endolysins LysAm24, LysAp22, LysECD7, and LysSi3: breakdown the matrix. World J Microbiol Biotechnol 40:186. 10.1007/s11274-024-03999-9 38683213 10.1007/s11274-024-03999-9

[45] Golban M, Charostad J, Kazemian H, Heidari H (2024) Phage-Derived Endolysins Against Resistant Staphylococcus spp.: A Review of Features, Antibacterial Activities, and Recent Applications. Infect Dis Ther. 10.1007/s40121-024-01069-z10.1007/s40121-024-01069-z 39549153 10.1007/s40121-024-01069-z PMC11782739

[46] Behera M, De S, Ghorai SM (2024) The Synergistic and Chimeric Mechanism of Bacteriophage Endolysins: Opportunities for Application in Biotherapeutics, Food, and Health Sectors. Probiotics Antimicrob Proteins. 10.1007/s12602-024-10394-110.1007/s12602-024-10394-1 39508962 10.1007/s12602-024-10394-1

[47] McCallin S, Drulis-Kawa Z, Ferry T, Pirnay JP, Nir-Paz R, antibacterials EEsgfn‑t (2023) Phages and phage-borne enzymes as new antibacterial agents. Clin Microbiol Infect. 10.1016/j.cmi.2023.10.018 37866680 10.1016/j.cmi.2023.10.018

[48] Khan FM, Chen JH, Zhang R, Liu B (2023) A comprehensive review of the applications of bacteriophage-derived endolysins for foodborne bacterial pathogens and food safety: recent advances, challenges, and future perspective. Front Microbiol 14:1259210. 10.3389/fmicb.2023.1259210 37869651 10.3389/fmicb.2023.1259210 PMC10588457

[49] Abdelrahman F, Easwaran M, Daramola OI et al (2021) Phage-Encoded Endolysins. Antibiot. 10.3390/antibiotics10020124 10.3390/antibiotics10020124 PMC7912344 33525684

[50] Gondil VS, Harjai K, Chhibber S (2020) Endolysins as emerging alternative therapeutic agents to counter drug-resistant infections. Int J Antimicrob Agents 55:105844. 10.1016/j.ijantimicag.2019.11.001 31715257 10.1016/j.ijantimicag.2019.11.001

[51] Briers Y, Walmagh M, Grymonprez B et al (2014) Art-175 is a highly efficient antibacterial against multidrug-resistant strains and persisters of Pseudomonas aeruginosa. Antimicrob Agents Chemother 58:3774–3784. 10.1128/AAC.02668-14 24752267 10.1128/AAC.02668-14 PMC4068523

[52] Sanchez-Torres V, Kirigo J, Wood TK (2024) Implications of lytic phage infections inducing persistence. Curr Opin Microbiol 79:102482. 10.1016/j.mib.2024.102482 38714140 10.1016/j.mib.2024.102482

[53] Stojowska-Swędrzyńska K, Kuczyńska-Wiśnik D, Laskowska E (2023) New Strategies to Kill Metabolically-Dormant Cells Directly Bypassing the Need for Active Cellular Processes. Antibiot. 10.3390/antibiotics12061044 10.3390/antibiotics12061044 PMC10295454 37370363

[54] Antonova NP, Vasina DV, Grigoriev IV et al (2024) Pharmacokinetic and preclinical safety studies of endolysin-based therapeutic for intravenous administration. Int J Antimicrob Agents 64:107328. 10.1016/j.ijantimicag.2024.107328 39244166 10.1016/j.ijantimicag.2024.107328

[55] Antonova NP, Vasina DV, Grigoriev IV et al (2024) Pharmacokinetics and Preclinical Safety Studies of Modified Endolysin-based Gel for Topical Application. J Pharm Sci 113:2093–2100. 10.1016/j.xphs.2024.04.028 38692487 10.1016/j.xphs.2024.04.028

[56] Zhang L, Li D, Li X et al (2016) LysGH15 kills Staphylococcus aureus without being affected by the humoral immune response or inducing inflammation. Sci Rep 6:29344. 10.1038/srep29344 27385518 10.1038/srep29344 PMC4935890

[57] Fowler VG Jr., Das AF, Lipka-Diamond J et al (2020) Exebacase for patients with Staphylococcus aureus bloodstream infection and endocarditis. J Clin Invest 130:3750–3760. 10.1172/JCI136577 32271718 10.1172/JCI136577 PMC7324170

[58] Fowler VG Jr., Das AF, Lipka-Diamond J et al (2024) Exebacase in Addition to Standard-of-Care Antibiotics for Staphylococcus aureus Bloodstream Infections and Right-Sided Infective Endocarditis: A Phase 3, Superiority-Design, Placebo-Controlled, Randomized Clinical Trial (DISRUPT). Clin Infect Dis 78:1473–1481. 10.1093/cid/ciae043 38297916 10.1093/cid/ciae043

[59] Moreau M, Seité S, Aguilar L et al (2021) Topical S. aureus-Targeting Endolysin Significantly Improves Symptoms and QoL in Individuals With Atopic Dermatitis. J Drugs Dermatol 20:1323–1328. 10.36849/jdd.6363 34898160 10.36849/jdd.6363

[60] Totté JEE, van Doorn MB, Pasmans S (2017) Successful Treatment of Chronic Staphylococcus aureus-Related Dermatoses with the Topical Endolysin Staphefekt SA.100: A Report of 3 Cases. Case Rep Dermatol 9:19–25. 10.1159/000473872 28611631 10.1159/000473872 PMC5465516

[61] Ferry T, Batailler C, Souche A et al (2021) Arthroscopic “Debridement and Implant Retention” With Local Administration of Exebacase (Lysin CF-301) Followed by Suppressive Tedizolid as Salvage Therapy in Elderly Patients for Relapsing Multidrug-Resistant S. epidermidis Prosthetic Knee Infection. Front Med 8:550853. 10.3389/fmed.2021.550853 10.3389/fmed.2021.550853 PMC8163228 34055817

[62] Pottie I, Vázquez Fernández R, Van de Wiele T, Briers Y (2024) Phage lysins for intestinal microbiome modulation: current challenges and enabling techniques. Gut Microbes 16:2387144. 10.1080/19490976.2024.2387144 39106212 10.1080/19490976.2024.2387144 PMC11305034

[63] Bratkovič T, Zahirović A, Bizjak M, Rupnik M, Štrukelj B, Berlec A (2024) New treatment approaches for Clostridioides difficile infections: alternatives to antibiotics and fecal microbiota transplantation. Gut Microbes 16:2337312. 10.1080/19490976.2024.2337312 38591915 10.1080/19490976.2024.2337312 PMC11005816

[64] Wilkinson HN, Stafford AR, Rudden M et al (2024) Selective Depletion of Staphylococcus aureus Restores the Skin Microbiome and Accelerates Tissue Repair after Injury. J Invest Dermatol 144:1865–1876 e1863. 10.1016/j.jid.2024.01.018 38307323 10.1016/j.jid.2024.01.018

[65] Totté J, de Wit J, Pardo L, Schuren F, van Doorn M, Pasmans S (2017) Targeted anti-staphylococcal therapy with endolysins in atopic dermatitis and the effect on steroid use, disease severity and the microbiome: study protocol for a randomized controlled trial (MAAS trial). Trials 18:404. 10.1186/s13063-017-2118-x 28859690 10.1186/s13063-017-2118-x PMC5580294

[66] Fenton M, Casey PG, Hill C et al (2010) The truncated phage lysin CHAP_k_ eliminates Staphylococcus aureus in the nares of mice. Bioeng Bugs 1:404–407. 10.4161/bbug.1.6.13422 21468207 10.4161/bbug.1.6.13422 PMC3056090

[67] Idelevich EA, Walther T, Molinaro S et al (2014) Bacteriophage-based latex agglutination test for rapid identification of Staphylococcus aureus. J Clin Microbiol 52:3394–3398. 10.1128/JCM.01432-14 25031449 10.1128/JCM.01432-14 PMC4313167

[68] Kretzer JW, Schmelcher M, Loessner MJ (2018) Ultrasensitive and Fast Diagnostics of Viable Listeria Cells by CBD Magnetic Separation Combined with A511::luxAB Detection. Viruses. 10.3390/v10110626 30428537 10.3390/v10110626 PMC6266503

[69] Hyeon SH, Lim WK, Shin HJ (2021) Novel surface plasmon resonance biosensor that uses full-length Det7 phage tail protein for rapid and selective detection of Salmonella enterica serovar Typhimurium. Biotechnol Appl Biochem 68:5–12. 10.1002/bab.1883 31916280 10.1002/bab.1883

[70] Cho JH, Kwon JG, O’Sullivan DJ, Ryu S, Lee JH (2021) Development of an endolysin enzyme and its cell wall-binding domain protein and their applications for biocontrol and rapid detection of Clostridium perfringens in food. Food Chem 345:128562. 10.1016/j.foodchem.2020.128562 33189482 10.1016/j.foodchem.2020.128562

[71] Nazir A, Xu X, Liu Y, Chen Y (2023) Phage Endolysins: Advances in the World of Food Safety. Cells. 10.3390/cells12172169 37681901 10.3390/cells12172169 PMC10486871

[72] Liu H, Kheirvari M, Tumban E (2023) Potential Applications of Thermophilic Bacteriophages in One Health. Int J Mol Sci. 10.3390/ijms24098222 37175929 10.3390/ijms24098222 PMC10179064

[73] Wire MB, Jun SY, Jang IJ, Lee SH, Hwang JG, Huang DB (2022) A Phase 1 Study To Evaluate Safety and Pharmacokinetics following Administration of Single and Multiple Doses of the Antistaphylococcal Lysin LSVT-1701 in Healthy Adult Subjects. Antimicrob Agents Chemother 66:e184221. 10.1128/AAC.01842-21 35007129 10.1128/aac.01842-21 PMC8923190

[74] Jun SY, Jang IJ, Yoon S et al (2017) Pharmacokinetics and Tolerance of the Phage Endolysin-Based Candidate Drug SAL200 after a Single Intravenous Administration among Healthy Volunteers. Antimicrob Agents Chemother. 10.1128/AAC.02629-16 28348152 10.1128/AAC.02629-16 PMC5444177

[75] de Wit J, Totte JEE, van Mierlo MMF et al (2019) Endolysin treatment against Staphylococcus aureus in adults with atopic dermatitis: A randomized controlled trial. J Allergy Clin Immunol 144:860–863. 10.1016/j.jaci.2019.05.020 31145938 10.1016/j.jaci.2019.05.020

